# Documenting symptom and context changes in individual case safety reports: a methodological perspective

**DOI:** 10.3389/fdsfr.2026.1885733

**Published:** 2026-10-28

**Authors:** Eik Niederlohmann

**Affiliations:** Department of Psychosomatic Medicine and Psychotherapy, Kliniken Erlabrunn, Breitenbrunn, Germany

**Keywords:** pharmacovigilance, individual case safety reports, adverse drug reaction reporting, case narratives, data quality, symptom trajectories, contextual information, follow-up

## Abstract

Individual case safety report narratives support pharmacovigilance by preserving clinical chronology and contextual information. A possible information gap concerns how documented symptom changes, surrounding circumstances, and actions already taken are linked and attributed to their sources. Its frequency and consequences have not been established. This Perspective proposes a minimal representation of symptom trajectories, context, actions, subsequent responses, concurrent changes, and remaining uncertainty. A limited procedural inspiration is the repeated observation and revision of working hypotheses used in dynamic psychotherapy; this does not establish a new pharmacovigilance diagnostic method. The proposal builds on existing case processing, causality frameworks, quality assessment, and follow-up practice. Evaluation would first audit source records and processing histories, then compare the proposed representation with optimized routine narratives containing the same facts. An independently adjudicated reference would specify what the records support, without claiming to establish biological causality. The primary endpoint would be correct reconstruction of prespecified case information; unsupported conclusions, completion, and workload would be assessed separately. Additional questioning would require a later, distinct evaluation of information yield, nonresponse, recall, and questioning effects. Contextual information must not delay reporting or justify unsupported dismissal of a medicinal-product contribution. The representation warrants testing, including the possibility that good routine processing already provides equivalent practical information.

## 1 Introduction: the proposal and its question

Individual case safety reports (ICSRs) support the assessment of suspected adverse reactions to medicinal products. Their narratives help pharmacovigilance (PV) assessors reconstruct the clinical course and identify questions requiring clarification. The issue considered here is whether available accounts of symptom and context changes can be represented more usefully. Neither the frequency of a specific documentation gap nor a resulting systematic causal error is established by the evidence reviewed.

The practical proposal is to preserve six connected elements: each symptom’s course, explicitly documented context, an action or situation change that already occurred, the subsequent response, concurrent changes, and remaining uncertainty. For example, a record might describe dizziness and palpitations after dosing, several actions during a telephone call, and improvement in only one symptom. The documentation should preserve that sequence and distinguish a contemporaneous report from a later recollection. It should not replace either account with an inferred psychological explanation.

The question is whether making these relationships explicit improves reconstruction compared with well-performed routine processing. Both representations must contain the same medical and contextual facts, sources, and uncertainties. The proposed addition would be redundant wherever routine narratives already make the relevant relationships sufficiently clear. Evaluation therefore needs to test an incremental information benefit rather than assume one from a visually structured example. This Perspective develops the representation and a staged research approach. It reports no empirical evaluation and proposes no new regulatory data requirement.

The proposed gap is relational: individually present details may be difficult to connect to a particular symptom, time, or source. Conversely, an explicit sequence can preserve ambiguity when the documents do not support a reliable connection. Success would mean recovering what is documented more faithfully, including its limits, rather than producing a more confident account.

## 2 What pharmacovigilance already captures

Routine PV already accommodates chronology, clinical course, concomitant treatment, dechallenge, and rechallenge. The International Council for Harmonisation (ICH) E2B (R3) standard for electronic transmission of ICSRs provides narrative and assessment fields alongside structured data (ICH, 2025a). The proposal does not introduce temporal reasoning into an otherwise static system. Nor does adding narrative headings demonstrate that readers will make better decisions.

Reporting conventions also matter. ICH E2D (R1) distinguishes spontaneous reports, which imply causal suspicion for reporting purposes, from solicited reports requiring a causality assessment (ICH, 2025b). Suspicion is not proof of causation. Organized collection, including patient support programs, does not by itself guarantee complete or unbiased information. These source types should not be pooled as interchangeable observations in an initial evaluation.

The World Health Organization (WHO) already addresses context in vaccine safety. Its causality-assessment manual for adverse events following immunization, in the 2019 update, recognizes developments concerning immunization stress-related responses (World Health Organization, 2021). The dedicated manual describes a broader range of stress-related responses than anxiety alone (World Health Organization, 2019). These are important precedents, not validation of the present representation for general medicine reports. The narrower contribution proposed here is an explicit, source-attributed description of documented changes, tested against existing competent practice.

Quality and reporting research further constrain the claim. VigiGrade evaluates documentation completeness, not whether a suspected reaction was actually caused by a medicine (Bergvall et al., 2014). Longitudinal presentation has also been developed for reviewing adverse drug events in clinical records (Geva et al., 2020). Follow-up questionnaires are established tools: European Medicines Agency guidance addresses specific questionnaires requested by regulators and emphasizes proportionate information collection (European Medicines Agency, 2024). A recent qualitative study of ten experts likewise supports targeting consequential information gaps, but does not quantify the benefit of this proposal (Kara et al., 2026). These approaches address different tasks. The proposed reconstruction test would complement completeness assessment by asking whether a reader can recover the documented relationships. It would not replace a quality instrument or establish a universal follow-up requirement.

Five processes should remain distinct: symptom occurrence, perception and reporting, information collection and processing, subsequent reconstruction or classification, and causal or signal assessment. Stimulated reporting concerns changes in reporting activity; notoriety bias can affect reporting patterns after safety alerts (Pariente et al., 2007). Expectancy-related symptoms, omitted narrative information, misclassification, and causal error are different problems. Their direction and magnitude require appropriate reference information and designs. Disproportionality measures are sensitive to several such mechanisms and do not establish incidence or individual causality (Fusaroli et al., 2026). A clearer narrative cannot be assumed to correct population-level reporting selection.

## 3 A dynamic principle, with a limited transfer

One procedural inspiration comes from intensive short-term dynamic psychotherapy (ISTDP). Its psychodiagnostic approach uses repeated observation of responses to interventions to revise working hypotheses and guide subsequent actions (Abbass and Haghiri, 2025; Michal et al., 2025). The transferable proposal here is limited to documenting sequences of symptoms, actions already taken, and subsequent changes. It does not ask PV personnel to apply psychotherapeutic pressure or challenge. Retrospective accounts remain recollections, not moment-to-moment observations. The clinical concept of a therapeutic window does not establish a validated PV assessment window; suitable conditions for any later information collection would need separate evaluation. This procedural origin neither validates psychological attribution of adverse events nor demonstrates diagnostic superiority in PV.

A dynamic description records relationships rather than merely adding labels such as “stress present.” It asks what was reported before and after an action, which symptoms changed, which persisted, and what else changed simultaneously. Repeated updates may revise the account as further evidence becomes available. An unchanged symptom is informative documentation, while an undocumented response is unknown. Neither should disappear when a summary emphasizes improvement. For case assessment, the resulting sequence could help locate a contradiction or identify a useful clarification: for example, whether the two symptoms actually followed different courses. An initially plausible interpretation may then remain open or require revision. These are proposed uses of the information, not demonstrated outcomes.

The distinction is especially important when several actions occur together. Less dizziness after sitting down, drinking water, and moving to a quieter room cannot isolate the contribution of any one change. Elapsed time, the medicine, other clinical processes, and contextual influences remain compatible explanations. A recorded temporal relationship is therefore a reconstruction target, not a causal test. Even accurate reporting of improvement does not establish why it occurred. The value of preserving the sequence must be demonstrated through readers' performance, including their ability to retain uncertainty and recognize unresolved medical questions.

## 4 A minimal representation in existing narratives

Table 1 specifies the six documentation elements. A trained case processor would assemble them from available intake records and follow-up material after necessary triage, or in parallel with timely reporting. The module is a proposed documentation structure, not a validated questionnaire or score. Context documentation must not delay acute care or required reporting, and these elements are not additional conditions for accepting a report.

**TABLE 1:** A minimal source-attributed representation of documented changes.

Documentation element	Neutral example	Information made explicit	Limit on interpretation
Symptom trajectory	Dizziness less intense; palpitations unchanged.	Separate course for each symptom.	Improvement in one symptom is not resolution of the event.
Documented context	Next-day recollection: a noisy room at onset.	Circumstance and retrospective source.	A recalled setting is not an observed mechanism.
Action or situation change	Patient reports sitting down, drinking water, and moving rooms.	Actions already taken and their sequence.	No action is assigned for diagnostic provocation.
Subsequent response	Initially unchanged; later, less dizziness.	Changed, unchanged, or unknown course.	A temporal sequence does not establish causation.
Concurrent changes	Posture, surroundings, and elapsed time changed together.	Other changes accompanying the reported response.	Their separate contributions cannot be isolated.
Remaining uncertainty	Pulse and later course not documented.	Missing or conflicting information requiring consideration.	Not documented does not mean normal, absent, or denied.

For each element, preserve source identity and location, source actor, and the available precision of event and recording times; retain later processing separately. Distinguish direct observations, contemporaneous patient or third-party reports, and later recollections. Entries describe existing records and do not require additional patient questioning. The elements can be documented in the factual narrative field H.1 of the International Council for Harmonisation E2B (R3) standard, with reporter and sender interpretations distinguished in H.2 and H.4 (ICH, 2025a). Unknown elements do not delay reporting. Examples are synthetic and illustrate documentation, not diagnostic rules.

Each statement should retain its source identifier and location, the person providing the information, and the distinction between event time, original recording time, and later processing. Precision should not exceed the source: “after a few minutes” must not become a timestamp. A clinician’s measurement, a contemporaneous patient or third-party report, and a later recollection are different kinds of evidence. Unknown information, an explicitly denied feature, and a reported absence of change require separate descriptions. Conflicting accounts should remain attributable rather than being silently reconciled. Reporters would not be expected to learn the module or classify mechanisms. Case processors would need shared worked examples and rules for preserving ambiguity; a clinical assessor would still decide whether unresolved information warrants ordinary medical follow-up.

The factual sequence can be represented in E2B (R3) H.1, while reporter and sender interpretations remain distinguishable in H.2 and H.4; H.3.r accommodates sender diagnosis or reclassification (ICH, 2025a). This field mapping concerns technical accommodation, not regulatory endorsement of the proposed structure. Changes to coding or assessment should follow applicable procedures, with the supporting evidence and reason recorded. The module must not turn an inferred mechanism into a source fact or obscure a reporter’s causal suspicion.

Figure 1 illustrates the intended contrast using an entirely synthetic case. Optimized routine narrative B and explicit representation C share all facts. During a same-day call, the person reports initially unchanged dizziness and palpitations after several actions, followed by less dizziness but persistent palpitations. A noisy setting at onset is recalled only the next day. Measurements and later clinical outcomes remain undocumented in both versions. B is deliberately coherent; C adds organization rather than privileged clinical information. The shared reconstruction task asks which symptom improved and which remained unchanged; the later course of palpitations remains an open follow-up question. The illustration establishes neither an information benefit nor the cause of the symptoms. In a real case, unresolved medical concerns would continue to guide appropriate assessment and follow-up.

**FIGURE 1:**
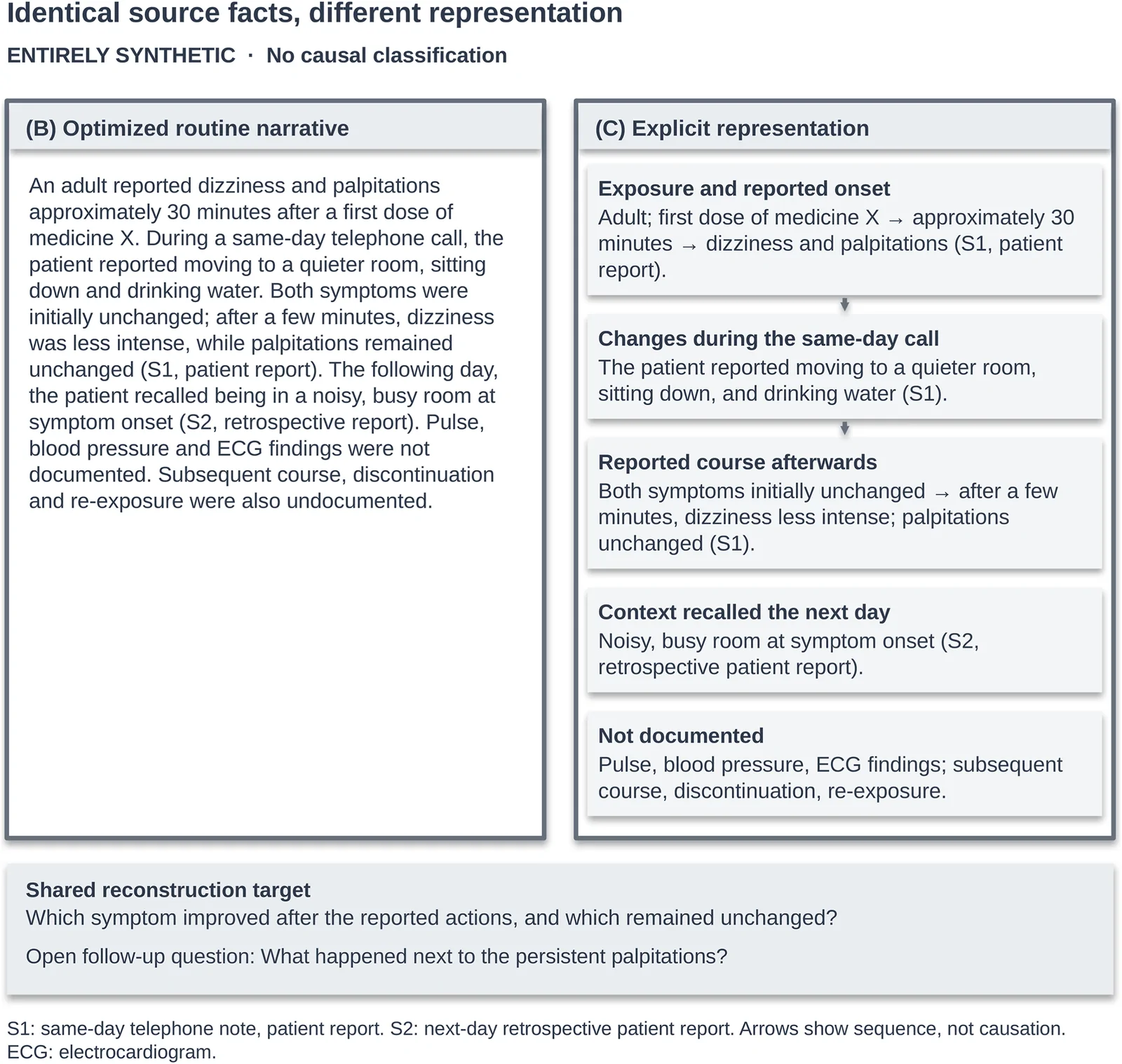
Synthetic example: identical source facts, different representation. Entirely synthetic example illustrating a representation contrast, not a demonstrated benefit. **(B)** is an optimized routine narrative; **(C)** explicitly links the same source-supported facts. S1 denotes a same-day telephone note containing patient reports, not direct clinical observations. S2 denotes a next-day retrospective account. Both representations preserve approximate timing, initially unchanged symptoms, later improvement in dizziness with persistent palpitations, and undocumented measurements and subsequent course. Temporal arrows indicate reported sequence, not causation. Several changes occur together; neither their individual effects nor a medicinal-product contribution can be determined. The shared reconstruction target concerns symptom-specific change; the open follow-up question concerns later palpitations. ECG, electrocardiogram.

## 5 Testing incremental information value

Evaluation should separate an audit of existing information, a controlled representation test, and any later intervention to collect additional information. A defensible first audit could sample adult spontaneous reports involving dizziness, palpitations, or dyspnea in a specified non-vaccine medicine setting and time period. Eligibility would include serious and nonserious reports without selecting presumed psychological cases. Random sampling should use case families, linking follow-ups and duplicates. Missing or inaccessible source records must remain visible in the sampling account.

Two independent coders would inventory relevant medical and contextual information in initial reports, actual follow-up records, and processing histories before examining final narratives. A third assessor would adjudicate disagreements; pre-adjudication agreement would also be reported. Audit outcomes would include the proportion of sampled case families with an assessable source-to-narrative chain and, within comparable chains, the proportion of relevant source facts omitted or altered. A final narrative alone cannot establish whether information was never requested, never received, or lost during processing.

The representation test would compare optimized routine narrative B with explicit representation C using the same source packet and factual content, including contextual details. Parity includes documented relationships, source attributions, and uncertainty, not merely isolated facts. Independent PV reviewers would check routine quality and factual parity before testing. Naturally clear relationships must not be removed from B to favor C. Length, layout, and preparation effort should be recorded, because the contrast evaluates a representation package rather than a pure effect of headings.

Assessors independent of the format authors would establish an adjudicated facts reference from the source packet before B and C were constructed. It would define what the records support, including source attribution, uncertainty, contradictions, and timing precision; it would not establish biological truth. Prespecified questions would address ordinary case-assessment needs, covering core medical facts as well as contextual relationships. Targets would be set independently of compliance with the proposed six-element structure. The scoring rubric would accept semantically equivalent answers, be pretested on separate cases, and be frozen before evaluation. It is a new measurement proposal requiring evaluation, not an already validated instrument.

Case-reader assignments would be randomized and balanced so that multiple readers assess each case in each format, while individual readers assess different cases in both formats. No reader would see two versions of the same case or linked case family. Training would be identical and order counterbalanced. Readers cannot be blinded to visible formatting; scoring of their answers should be masked to allocation where practicable. Cases used for training or rubric development would be excluded from testing.

The primary endpoint would be the proportion of prespecified factual targets correctly reconstructed in each case-reader assignment. The contrast would be C minus B, with cases weighted equally, reported in absolute percentage points with a confidence interval. Core medical targets would also be reported separately. Partial submissions retain credit for correct answers; unanswered targets and unstarted assignments contribute no successful reconstruction to this operational endpoint. Completion and accuracy among completed assignments would be reported separately. Technical data loss would be distinguished from noncompletion and examined through prespecified sensitivity analyses.

Analysis would account for both case and reader variation, following the general rationale for crossed random factors (Judd et al., 2012). Targets within a case should not be treated as independent case replications. Sample-size planning would require a prespecified practically relevant difference, desired precision, and plausible variance components. A nonsignificant result would not establish equivalence. Secondary outcomes would include unsupported factual additions, unwarranted definitive causal conclusions, justified clarification questions, inappropriate context-based dismissal, preparation time, active reading time, and completion. For each error type, the denominator would be assessable case-reader assignments, counting assignments with at least one error; unavailable responses would be reported separately. Agreement between readers would describe reproducibility, not causal accuracy.

Clarification performance would distinguish coverage of independently identified information gaps from unnecessary questions. Cases without such gaps would have no coverage denominator, but their unnecessary questions would still count. A separate decision task would assess whether context alone prompted unsupported dismissal or downclassification against prespecified processing rules. This would measure behavior in an offline task, not observed patient harm. Time logs would retain interrupted or aborted work. Research adjudication would be reported separately from the effort required to prepare and use a narrative in practice. Meaningful reconstruction gains, tolerable workload, and error limits would be prespecified before testing.

Only a later study should test additional questioning. It could randomize usual follow-up against usual follow-up plus neutral questions about what happened next, what changed or remained unchanged, and what else changed. Necessary clinical follow-up would remain available in both groups; output representation would be identical. Contact opportunities and time would be matched or their differences explicitly treated as part of the intervention. The study would report assigned cases, contact attempts, reach, response, partial response, and nonresponse. Additional source-attributed information and workload would be evaluated per assigned case, with responder-only results supplementary. New reports and processing delays would be tracked without presuming a desirable direction.

## 6 Discussion: feasibility, counterexamples and limits

The representation addresses processing and reconstruction. It cannot recover events that were never noticed or reported, and it creates no new information when source records are sparse. An audit might show that good routine processing already preserves the targeted relationships. Even when C improves reconstruction, additional preparation work or unsupported psychological conclusions could outweigh that gain. Such findings would argue against adoption or require redesign. A controlled reader experiment also does not establish effectiveness in routine workload conditions.

Active follow-up raises additional problems. Accounts may be selective, delayed, or shaped by hindsight. People who can be reached and are willing to discuss context may differ from nonresponders; observed response characteristics cannot exclude unmeasured selection. Retrospective timing should remain visibly approximate. Studies with prospectively collected clinical information may support richer assessment than routine spontaneous reports, but that advantage does not demonstrate routine feasibility. Expert perspectives on useful follow-up are valuable, while prospective information yield and burden remain empirical questions (Kara et al., 2026). The initial setting is consequently a bounded starting point, not a representative sample of all medicines, symptoms, reporting channels, or patient groups. Findings would need replication where source availability, language, and processing arrangements differ.

Questioning can itself change what is reported. In a randomized study during a placebo run-in, a symptom checklist elicited more adverse-event reports than open questions (Bent et al., 2006). This is evidence about ascertainment in a different setting, not a validation of the proposed questions or a determination of which reports were causally correct. Neutral wording and contact logs therefore cannot be assumed to eliminate questioning effects. More documented events could reflect greater ascertainment, altered reporting, or both.

Context must not become a shortcut for dismissing suspected reactions. Improvement after reassurance or a situation change does not exclude a medicinal-product contribution, and persistent symptoms require their own account. Legitimate evidence-based corrections remain possible, provided the original information, reasoning, and processing history are auditable. Neither reduced reporting volume nor unchanged seriousness distributions constitute a universal success criterion. Finally, shifts in causal ratings or signal measures require separate validation against an appropriate causal target; the facts reference proposed here cannot provide it. This Perspective’s literature assessment is narrative, and no prevalence estimate, implementation effect, or reduction in systematic causal error is claimed.

## 7 Conclusion

Source-attributed symptom and context sequences offer a specific documentation proposal that can be tested without introducing psychological diagnosis into PV intake. The immediate question is whether explicit representation helps readers reconstruct relevant information better than optimized routine narratives containing the same facts. A source audit and controlled comparison can test that limited claim. Adoption would additionally require acceptable workload and avoidance of unsupported dismissal; causal and signal benefits remain separate research questions.

## Data Availability

No datasets were generated or analyzed for this Perspective article. The examples in Table 1 and Figure 1 are entirely synthetic and are included in the article. Further inquiries can be directed to the corresponding author.
